# Plasma p-tau231: a new biomarker for incipient Alzheimer’s disease pathology

**DOI:** 10.1007/s00401-021-02275-6

**Published:** 2021-02-14

**Authors:** Nicholas J. Ashton, Tharick A. Pascoal, Thomas K. Karikari, Andréa L. Benedet, Juan Lantero-Rodriguez, Gunnar Brinkmalm, Anniina Snellman, Michael Schöll, Claire Troakes, Abdul Hye, Serge Gauthier, Eugeen Vanmechelen, Henrik Zetterberg, Pedro Rosa-Neto, Kaj Blennow

**Affiliations:** 1 Department of Psychiatry and Neurochemistry, Institute of Neuroscience and Physiology, The Sahlgrenska Academy at the University of Gothenburg, Mölndal, Sweden; 2 Wallenberg Centre for Molecular and Translational Medicine, University of Gothenburg, Gothenburg, Sweden; 3 King’s College London, Institute of Psychiatry, Psychology and Neuroscience, Maurice Wohl Institute Clinical Neuroscience Institute, London, UK; 4 NIHR Biomedical Research Centre for Mental Health and Biomedical Research Unit for Dementia at South London and Maudsley NHS Foundation, London, UK; 5 Translational Neuroimaging Laboratory, McGill Centre for Studies in Aging, McGill University, Montreal, QC Canada; 6 Department of Psychiatry and Neurology, University of Pittsburgh, Pittsburgh, PA USA; 7 Alzheimer’s Disease Research Unit, The McGill University Research Centre for Studies in Aging, Montreal, McGill University, Montreal, QC Canada; 8 ADx NeuroSciences, Technologiepark 94, 9052 Ghent, Belgium; 9 Clinical Neurochemistry Laboratory, Sahlgrenska University Hospital, Mölndal, Sweden; 10 Department of Neurodegenerative Disease, UCL Institute of Neurology, Queen Square, London, UK; 11 UK Dementia Research Institute at UCL, London, UK; 12 Montreal Neurological Institute, Montreal, QC Canada; 13 Department of Neurology and Neurosurgery, McGill University, Montreal, QC Canada; 14 Department of Basic and Clinical Neuroscience, Institute of Psychiatry, Psychology and Neuroscience, King’s College London, London, UK

**Keywords:** Alzheimer’s disease, Tau, Braak, Biomarkers, Blood, Preclinical, p-tau231, p-tau181, p-tau217

## Abstract

The quantification of phosphorylated tau in biofluids, either cerebrospinal fluid (CSF) or plasma, has shown great promise in detecting Alzheimer’s disease (AD) pathophysiology. Tau phosphorylated at threonine 231 (p-tau231) is one such biomarker in CSF but its usefulness as a blood biomarker is currently unknown. Here, we developed an ultrasensitive Single molecule array (Simoa) for the quantification of plasma p-tau231 which was validated in four independent cohorts (*n* = 588) in different settings, including the full AD continuum and non-AD neurodegenerative disorders. Plasma p-tau231 was able to identify patients with AD and differentiate them from amyloid-β negative cognitively unimpaired (CU) older adults with high accuracy (AUC = 0.92–0.94). Plasma p-tau231 also distinguished AD patients from patients with non-AD neurodegenerative disorders (AUC = 0.93), as well as from amyloid-β negative MCI patients (AUC = 0.89). In a neuropathology cohort, plasma p-tau231 in samples taken on avergae 4.2 years prior to post-mortem very accurately identified AD neuropathology in comparison to non-AD neurodegenerative disorders (AUC = 0.99), this is despite all patients being given an AD dementia diagnosis during life. Plasma p-tau231 was highly correlated with CSF p-tau231, tau pathology as assessed by [^18^F]MK-6240 positron emission tomography (PET), and brain amyloidosis by [^18^F]AZD469 PET. Remarkably, the inflection point of plasma p-tau231, increasing as a function of continuous [^18^F]AZD469 amyloid-β PET standardized uptake value ratio, was shown to be earlier than standard thresholds of amyloid-β PET positivity and the increase of plasma p-tau181. Furthermore, plasma p-tau231 was significantly increased in amyloid-β PET quartiles 2–4, whereas CSF p-tau217 and plasma p-tau181 increased only at quartiles 3–4 and 4, respectively. Finally, plasma p-tau231 differentiated individuals across the entire Braak stage spectrum, including Braak staging from Braak 0 through Braak I–II, which was not observed for plasma p-tau181. To conclude, this novel plasma p-tau231 assay identifies the clinical stages of AD and neuropathology equally well as plasma p-tau181, but increases earlier, already with subtle amyloid-β deposition, prior to the threshold for amyloid-β PET positivity has been attained, and also in response to early brain tau deposition. Thus, plasma p-tau231 is a promising novel biomarker of emerging AD pathology with the potential to facilitate clinical trials to identify vulnerable populations below PET threshold of amyloid-β positivity or apparent entorhinal tau deposition.

**Supplementary Information:**

The online version contains supplementary material available at 10.1007/s00401-021-02275-6.

## Introduction

The aberrant accumulation of aggregated amyloid-β (Aβ) peptides and abnormally phosphorylated tau protein as extracellular plaques and intraneuronal neurofibrillary tangles (NFTs), respectively, are the fundamental hallmarks of Alzheimer’s disease (AD) neuropathology and remain to be the conclusive confirmation of the disease—thus a definitive diagnosis cannot be attributed until post-mortem. Nonetheless, the characterization of AD in vivo has been greatly enhanced by the visualization of Aβ [52] and tau [35] aggregates by positron emission tomography (PET). The reduction of Aβ42 or Aβ42/40 and the increase of total-tau (t-tau) and phosphorylated tau (p-tau) represent the core cerebrospinal fluid (CSF) biomarker changes [43]. However, both CSF and PET biomarkers have significant practical, logistic and economical drawbacks and are unlikely to be widely used in primary care for the assessment of AD or other neurodegenerative diseases. Furthermore, given recent clinical trial failures, confirmatory evidence of underlying pathology is being more commonly required as a fundamental selection criterion for AD therapeutic trials. Therefore, the field would greatly benefit from a more easily implemented and cost-effective approach to the initial patient or participant assessment that could satisfy both clinical and drug development needs.

The emergence of blood biomarkers for the specific detection of AD pathophysiology offers the scalability required for patient or population triage. This would complement CSF and PET biomarkers in the initial assessment in memory clinics or therapeutic trial recruitment and monitoring. Blood neurofilament light (NfL), a biomarker of axonal injury, is robustly increased in AD [5, 37] but is also increased in many other neurodegenerative disorders [4] and upon acute neurological injury [64]. Mass spectrometric assays for plasma Aβ42 or Aβ42/40 have demonstrated high accuracy in detecting cerebral Aβ pathology [45, 56]. However, the fold-change of Aβ42/40 peptides, between Aβ-positive and Aβ-negative individuals, is substantially reduced in plasma than CSF—likely owing to peripheral expression of Aβ. Therefore, plasma NfL and Aβ do not have the required specificity to routinely detect underlying AD pathology. P-tau, however, is a highly specific pathological biomarker of AD and the diagnostic capabilities of CSF p-tau at threonine 181 (p-tau181) [12] have been widely replicated in blood. Plasma p-tau181 can differentiate AD from non-AD neurodegenerative disorders [10, 25, 27, 29, 34, 60], detect AD neuropathology [25, 34, 60], identifies individuals with increased Aβ and tau PET retention [25, 27, 29, 41, 60], strongly associates with imminent grey matter atrophy [57] and predicts progression to AD dementia, to the same degree as CSF p-tau181 [25, 27, 44]. Furthermore, findings of CSF p-tau at threonine 217 (p-tau217), which suggests stronger associations with AD pathology than standard CSF p-tau181 biomarkers [6, 26, 28], have also been replicated in plasma [38, 48]. Therefore, plasma p-tau181 and p-tau217 are highly promising clinical biomarkers in the assessment of memory complaints and risk of disease progression.

The presence of p-tau in biofluids has been proposed to be a cellular response to NFT pathology [23]. However, the increases of CSF p-tau residues precede detectable NFT pathology, indexed by tau PET, by up to a decade [8, 62], and plasma p-tau181 is already increased in tau PET-negative but Aβ PET-positive individuals [29]. This demonstrates that soluble tau, in CSF and plasma, is likely indicative of early tau pathology which is closely correlated with Aβ deposition. We have recently described the increase of different phosphoforms of tau in CSF in preclinical and prodromal disease [3, 28, 58]. In two independent studies [3, 58], we found that CSF p-tau phosphorylated at threonine 231 (p-tau231) is a biomarker of very early tau pathology. CSF p-tau231 begins to increase concurrently with Aβ pathology in preclinical disease [58] and in Aβ-negative CU individuals shows focal associations with emerging Aβ pathology in the medial orbitofrontal, precuneus and posterior cingulate cortices [3]. To this end, given the successful translation of the CSF p-tau181 and CSF p-tau217 assays to blood tests, we hypothesized that the detection of p-tau231 in plasma would be possible, and further, would reflect the advantageous characteristics of CSF p-tau231 being a biomarker for very early AD pathology, which begins to increase before Aβ PET positivity is achieved. Such a biomarker could be utilized as a simple and cost-effective biomarker to detect preclinical or even earlier emerging pathology prior to substantial Aβ accumulation for therapeutic trials recruitment and would be highly complementary to the characteristics of the plasma p-tau181 and p-tau217 markers.

In this study, we report the first ultrasensitive immunoassay for the precise quantification of plasma p-tau231. We assessed the capabilities of this novel blood biomarker in the detection of in vivo AD pathophysiology, specifically; (i) the differentiation of AD from cognitively healthy individuals and other neurodegenerative diseases, including neuropathological confirmation; (ii) the segregation of individuals across the AD continuum, (iii) use as a primary care biomarker and (iv) the determination of forthcoming cognitive decline and hippocampal atrophy. Further, given our recent findings for CSF p-tau231, we also assessed if plasma p-tau231 could detect early abnormalities in amyloid-β or tau PET scans, with a focus on preclinical disease and in comparison to p-tau181 and p-tau217 epitopes.

## Methods

### Sample cohorts

The discovery cohort included plasma from biochemically defined AD patients (*n* = 20) and age-matched controls (*n* = 18). The AD patients were clinically assessed for suspected AD and demonstrated no evidence of other neurological conditions (*e.g.*, co-existing inflammatory or cerebrovascular disease). AD patients demonstrated a typical AD CSF biomarkers profile; CSF Aβ42 < 530 ng/L, CSF p-tau > 60 ng/L, and CSF t-tau > 350 ng/L. The control group consisted of patients with minor neurological or psychiatric symptoms, and with core CSF biomarker levels within normal ranges. The use of these patient samples has been approved by the Ethics Committee at the University of Gothenburg (EPN 140811).

The discovery findings were independently replicated in the Translational Biomarkers of Aging and Dementia (TRIAD) cohort, McGill University, Canada [51] (*n* = 313). In the TRIAD cohort, participants had CSF and PET (amyloid-β and tau) biomarkers and detailed clinical and cognitive assessments, including Mini-Mental State Examination (MMSE) and the clinical dementia rating (CDR) tests. The TRIAD cohort consisted of cognitively unimpaired (CU) young (age range, 20–30 years) and CU elderly (age range, 50–86 years) as well as mild cognitive impairment (MCI), AD, and non-AD dementia patients. CU participants (*n* = 16) who were considered middle-age adults (age range, 30–50 years) were included in the CU elderly group for the analysis. CU participants had an MMSE score > 24 and a CDR score of 0. MCI participants had a CDR score of 0.5, subjective and objective impairments in cognition, but preserved activities of daily living. AD dementia patients had a CDR score ≥ 0.5 and met the National Institute on Aging and the Alzheimer’s Association criteria for probable Alzheimer’s disease determined by a physician [40]. The non-AD dementia participants had CDR score ≥ 0.5, were Aβ PET negative and had a clinical diagnosis of FTD (*n* = 10), primary progressive aphasia (*n* = 1), cortical basal degeneration (*n* = 1), progressive supranuclear palsy (*n* = 2), vascular cognitive impairment (*n* = 10), hippocampal sclerosis (*n* = 1), or cerebral amyloid angiopathy (*n* = 1). In the TRIAD cohort, participants were excluded if they had active substance abuse or inadequately treated conditions, recent head trauma or major surgery, or if they presented safety contraindication for the study procedures.

In the primary care setting cohort (*n* = 190) we aimed to evaluate the new blood biomarker in the real-life setting, where blood biomarkers were likely to have a significant role. We included controls from the community without a diagnosis of a neurological condition and patients referred to the McGill University Research Centre for Studies in Aging memory clinic, Canada, from primary care physicians. These individuals had received a clinical diagnosis by the primary care physicians but were yet to undergo biomarker and clinical assessments in the specialized memory clinic setting. The TRIAD and the primary-care cohort were approved by the Douglas Mental Health University Institute Research Ethics Board and the Montreal Neurological Institute PET working committee, and a written informed consent was obtained for all participants.

The neuropathology cohort (*n* = 47) aimed to evaluate the specificity of p-tau231 in cases with a definite diagnosis of AD against other neurodegenerative disorders with a definite diagnosis (not "only" clinical evaluations and PET scans as for the validation and primary care cohorts). We included AD and non-AD neuropathologically confirmed cases who provided a plasma sample 1–9 years (mean = 4.2 years) prior to death. At the time of clinical assessment, a diagnosis of probable or possible AD (AD dementia) was made according to the Diagnostic and Statistical Manual for Mental Diagnosis, fourth edition and National Institute of Neurological, Communicative Disorders and Stroke–Alzheimer’s disease and Related Disorders Association (NINCDS-ADRDA) clinical criteria [39]. No CSF or imaging assessments were performed for these individuals. Consent for autopsy, neuropathological assessment and research was obtained for all cases and the study was carried out under the ethical approval of the Medical Research Council (MRC) London Neurodegenerative Diseases Brain Bank, Institute of Psychiatry, King’s College London. Block taking and neuropathological assessment was performed according to standard criteria for the diagnosis of neurodegenerative disease. Assessments included Braak staging for NFT [13] and reporting of co-existing pathology such as cerebrovascular lesions, TAR DNA-binding protein 43 (TDP-43), and Lewy body pathology. Non-AD dementias had no or sparse neurite plaque pathology and were classified as progressive supranuclear palsy (*n* = 3), frontotemporal lobe degermation (*n* = 3), Lewy body dementia (*n* = 2), vascular dementia (*n* = 2), cerebral amyloid angiopathy (*n* = 1) by two independent pathologists.

### Phosphorylated tau blood measurements

All plasma p-tau biomarkers in this study were measured using *in-house* Single molecule array (Simoa) methods on the HD-X instrument (Quanterix) at the Clinical Neurochemistry Laboratory, Sahlgrenska University Hospital, Mölndal, Sweden. Plasma p-tau181 and plasma NfL measurements used in this study have been previously reported [9, 29].

For the novel plasma p-tau231 Simoa assay, monoclonal mouse antibodies were generated using a synthetic peptide (K_224_KVAVVR(pT)PPKSPSSAK_240_C) as a KLH-coupled antigen, numbered according to full-length tau-441 phosphorylated on threonine 231. Candidate hybridomas were selected on brain extracts of AD and control brain tissue. The final cloned and purified monoclonal antibody, ADx253, was characterized on synthetic peptides spanning amino acids threonine 217 till serine 241 of full-length tau for its affinity, its phospho-specificity using both phosphorylated and non-phosphorylated peptides and its preferred selectivity in which position 232 was replaced by a Pip, to simulate cis-selectivity of ADx253. A biotin-conjugated N-terminal anti-tau mouse monoclonal antibody was used for detection. Full-length recombinant tau 441 phosphorylated in vitro by glycogen synthase kinase 3β was used as the calibrator. The assay validation focused on dilution linearity, spike recovery, antibody specificity, precision and lower limit of quantification (LLOQ) are described in the supplementary methods, supplementary Table 1–3 and supplementary Fig. 1–2, online resource.

### CSF measurements

In the discovery cohort, CSF p-tau181, total tau and Aβ42 were measured using the established INNOTEST ELISA assays from Fujirebio. The fully automated LUMIPULSE G1200 (Fujirebio) was used to measure CSF p-tau181, total tau and Aβ42/Aβ40 for the validation and clinical cohorts. CSF p-tau217 and CSF p-tau231 were quantified by the custom Simoa and ELISA assays, respectively, and have been previously described in detail [28, 58].

### Imaging analysis

All individuals in the TRIAD cohort were assessed with Siemens 3 T MRI as well as Aβ [^18^F]AZD4694 PET and tau [^18^F]MK-6240 PET acquired with a Siemens High-Resolution Research Tomograph. [^18^F]MK-6240 images were acquired at 90–110 min after the intravenous bolus injection of the radiotracer [50, 51]. [^18^F]AZD4694 PET images were acquired at 40–70 min after the intravenous bolus injection of the radiotracer [50, 51]. The PET images were spatially smoothed to achieve a final 8-mm full width at half maximum resolution and were processed using a previously described pipeline [50, 51], [^18^F]MK6240 images were stripped off the meninges before smoothing, as described elsewhere [51]. [^18^F]MK-6240 SUVR was measured regionally in the transentorhinal (stage I–II), limbic (III–IV), and isocortical (V–VI) regions approximating Braak stages of NFT pathology, as previously described, and tau positivity was defined as 2.5 standard deviations (SD) higher than the mean SUVR of CU Aβ-negative elderly [29]. Individuals negative for tau PET uptake in all aforementioned regions-of-interest were classified as Braak stage 0. Global [^18^F]AZD4694 SUVR was derived from averaging retention in the precuneus, the cingulate, inferior parietal, medial prefrontal, lateral temporal, and orbitofrontal cortices. A [^18^F]AZD4694 SUVR positivity was determined at SUVR > 1.55 (centiloid [32] = 22), as detailed elsewhere [59]. The intracranial volume corrected hippocampal volume was measured with Freesurfer version 6.0 using the Desikan-Killiany-Tourville gray matter parcellation [31].

### Statistical analysis

The statistical analyses were performed using R statistical software version 3.1.2 (http://www.r-project.org/). The voxel-wise statistics were performed using MATLAB v9.2 using the VoxelStats package [36]. The group-wise comparisons were assessed using unpaired analysis of variance with Tukey’s multiple comparisons test at *P* < 0.05, whereas associations between biomarkers were tested with linear regressions or Spearman rank correlation analysis. Voxel-wise associations were performed with linear regression models false discovery rate corrected for multiple comparisons at *P* < 0.05. The receiver operating characteristic curve (ROC) comparing groups provided the area under the curve (AUC) for a clinical diagnosis. In addition, we modeled and plotted p-tau biomarker levels as a function of Aβ PET load using a local weighted regression model, as performed elsewhere [17, 42, 47]. In this analysis, plasma p-tau231 and p-tau181 biomarker values were corrected for age and sex and converted to z-scores anchored on the normative data of CU Aβ-negative. The associations between plasma p-tau231 concentrations and longitudinal changes in MMSE score and hippocampal volume were assessed with linear regression models. The longitudinal changes in MMSE score and hippocampal volume were calculated as the difference between follow-up and baseline values divided by time duration between the measurements.

## Results

### Development and performance of plasma p-tau231 single-molecule array (Simoa)

The plasma p-tau231 assay demonstrated high analytical performance (supplementary Table 1–2, online resource) with high precision within and between clinical studies (supplementary Table 3, online resource). Immunoprecipitation and mass spectrometric studies showed that the assay specifically measures N-terminal to mid-domain forms of tau phosphorylated at threonine-231 and does not recognize non-phosphorylated forms of tau (supplementary Fig. 1–2, online resource). For the determination of LLOQ, the calibrator was analysed in duplicates at concentrations between 64 and 0.25 pg/mL. The deviation of the signal-calculated concentrations from the known values was estimated. The LLOQ was set as the calibrator point before the coefficient of variance increased above 20%. In the clinical studies, 588 out of 597 (98.5%) samples measured above the assay LLOQ of 2 pg/ml. The 9 samples measuring below the LLOQ included Aβ-negative CU (*n* = 6) and non-AD cases (*n* = 3).

### Study participants

There were 38 individuals in the discovery cohort (supplementary table 4, online resource). We assessed a further 313 individuals from the TRIAD cohort (McGill University, Canada). This included young adults (*n* = 32), CU elderly (*n* = 189), MCI (*n* = 54), AD (*n* = 42) and non-AD neurodegenerative disorders (*n* = 26), which is displayed in Table 1. The primary care cohort (*n* = 190, Table 2) are individuals who have received a clinical diagnosis but were yet to undergo biomarker and clinical assessments in the specialized memory clinic setting. The neuropathology cohort (*n* = 47, Table 2) included individuals with a plasma sample obtained between 1 and 9 years (mean = 4.2 years) prior to *post-mortem* where a definite neuropathological diagnosis of either AD (*n* = 36) or non-AD dementia (*n* = 11).

**Table 1:** Characteristics of the TRIAD cohort

	TRIAD cohort (*n* = 313)				
	Young adults (*n* = 32)	CU elderly adults (*n* = 159)	MCI (*n* = 54)	AD (*n* = 42)	Non-AD (*n* = 26)
Age, years	22.8 (1.5) ^†^	69.2 (10.2)	69.8 (7.1)	65.7 (9.2)	66.7 (7.1) ^*^
Sex Women Men	20 (62.5%) 12 (37.5%)	101 (63.5%) 58 (36.5%)	29 (53.7%) 25 (46.3%)	20 (47.6%) 22 (52.4%)	12 (46.1%) 14 (45.1%)
*APOE* ε4 carriership	8 (25%) ^†^	45 (28.3%) ^†^	27 (50%)	29 (69.1%) ^*^	5 (19.2%) ^†^
MMSE score	29.8 (0.5) ^†^	29.1 (1.1) ^†^	27.8 (1.8) ^†*^	18.5 (5.7) ^*^	25.6 (9.7) ^†^
CSF Biomarkers (pg/mL) Aβ42 P-tau181 T-tau	828.7 (242.6)^†^ 22.7 (6.1) ^†^ 201.7 (51.2) ^†^	986.0 (432.2) ^†^ 43.3 (40.2) ^†^ 333.3 (122.2) ^†^	706.0 (345.3) ^†*^ 79.8 (59.1) ^†*^ 502.9 (295.7) ^†*^	439.0 (142.2) ^*^ 106.6 (39.6) ^*^ 700.4 (345.8) ^*^	930.3 (165.7) ^†^ 32.9 (9.2) ^†^ 311.9 (70.3) ^†^
Aβ PET SUVR % Aβ-positive	1.15 (0.2) ^†^ 0%	1.38 (0.4) ^†^ 19.4%	1.96 (0.6) ^†^ 79.6%	2.19 (0.5) ^*^ 100%	1.21 (0.1) ^†^ 0%
Tau PET SUVR Braak I-II Braak III-IV Braak V-VI	0.85 (0.3) ^†^ 0.91 (0.9) ^†^ 1.1 (0.2) ^†^	0.97 (0.2) ^†^ 0.95 (0.1) ^†^ 0.97 (0.1) ^†^	1.58 (0.6) ^†*^ 1.29 (0.5) ^†*^ 1.14 (0,5) ^†*^	1.98 (0.6) ^*^ 2.61 (1.1) ^*^ 2.25 (2.1) ^*^	0.87 (0.1) ^†*^ 0.91 (0.1) ^†^ 0.96 (0.1) ^†^
Plasma biomarkers (pg/mL) P-tau181 P-tau231 NfL	8.22 (2.6) ^†^ 9.41 (3.2) ^†^ 6.24 (1.3) ^†^	10.91 (3.3) ^†^ 14.94 (4.1) ^†^ 23.12 (2.2) ^†^	16.26 (6.7) ^†*^ 19.45 (7.1) ^†*^ 24.62 (8.2) ^†^	25.21 (7.8) ^*^ 29.22 (8.2) ^*^ 34.04 (8.3) ^*^	9.91 (2.1) ^†^ 11.53 (4.1) ^†^ 28.31 (7.2) ^*^

Data in mean (SD) or *n* (%). We used analysis of variance followed by Tukey’s post hoc test to assess differences between groups for continuous variables. For sex and *APOE* ε4 genotype, we used contingency *χ*^2^ tests. CU = cognitively unimpaired. MCI = mild cognitive impairment. MMSE = Mini-Mental State Examination. SUVR = standardised uptake value ratio. p-tau181 = tau phosphorylated at threonine 181. p-tau231 = tau phosphorylated at threonine 231. NfL = neurofilament light chain. *p < 0·05 compared with cognitively unimpaired older adults. †*p* < 0·05 compared with Alzheimer’s disease

**Table 2:** Characteristics of the primary care and neuropathology cohort

	Primary care cohort (*n* = 190)				Neuropathology (*n* = 47)	
	Young adults (*n* = 8)	CU elderly adults (*n* = 131)	MCI (*n* = 17)	AD (*n* = 34)	AD (*n* = 36)	Non-AD (n = 11)
Age, years	23.1 (1.2) ^*^	70.8 (8.1)	70.1 (7.2)	65.4 (13.2)	81.5 (17.5)	79.7 (10.9)
Sex Men Women	5 (62.5%) 3 (37.5%)	44 (33.5%) 87 (66.5%)	6 (35.3%) 11 (64.7%)	13 (38.2%) 21 (61.8%)	12 (33.3%) 24 (66.7%)	5 (45.5%) 6 (54.5%)
*APOE* ε4 carriership	3 (37.5%)	31 (23.6%)	6 (35.3%)	20 (58.8%	20 (55.6%)	2 (18.2%)
Education, years	18.8 (2.4)	14.1 (3.6)	13.0 (4.5)	8 (4.1) ^a^	31.5 (9.5) ^b^	13.2 (2.7)
Braak Score (I/II/III/IV/V/VI)	**…**	**…**	**…**	**…**	0/0/0/7/5/24	1/5/5/0/0/0
Time from plasma collection to post-mortem, years (range)	**…**	**…**	**…**	**…**	4.0 (0.8–8)	4.2 (2–9)

^a^*p* < 0.01 compared with cognitively unimpaired older adults

^b^*p* < 0.0001 compared with non-AD

### Plasma p-tau231 shows high performance for the diagnosis of AD

**Fig. 1:**
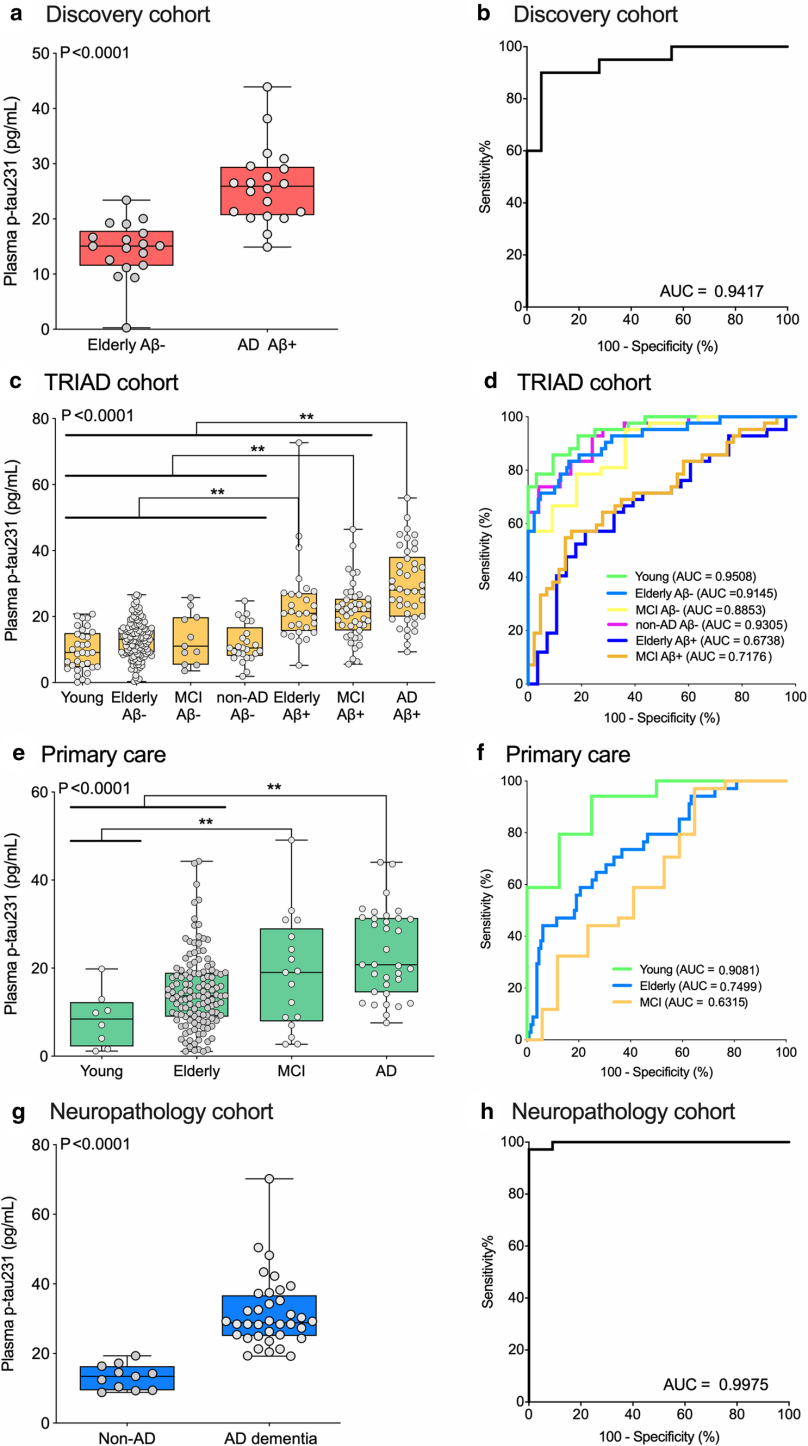
Plasma p-tau231 concentration identifies individuals across the AD spectrum and neuropathologically confirmed AD cases. The box-and-whisker plots (left side) show plasma phospho-tau231 (p-tau231) concentrations across groups. For the box-and-whisker plots, the horizontal bar shows the median, and the upper and lower boundaries show the 25th and 75th percentiles, respectively. *P* values indicate the results of the analysis of variance models with Tukey’s post hoc multiple comparisons at ** *P* < 0.05. The figure also displays the corresponding ROC curves in the four cohorts studied (right side) to separate individuals with AD dementia from the other groups. The AUC values of the ROC curves indicate the overall biomarker performance across groups, with 0.5 indicating no difference from chance and 1.0 a biomarker with specificity and sensitivity of 100%. **a**, **b** In the discovery cohort (*n* = 38), plasma p-tau231 concentrations accurately discriminated AD from CU elderly Aβ- controls. **c**, **d** In the TRIAD validation cohort (*n* = 313), AD dementia individuals had higher plasma p-tau231 levels than all other groups. **e**, **f** In the primary care clinical cohort (*n* = 190), AD dementia individuals had higher plasma p-tau231 than CU but not than MCI individuals. **g**, **h** In the neuropathology cohort (*n* = 47) clinical cohort, AD individuals had higher plasma p-tau231 than non-AD dementias

In the discovery cohort, plasma p-tau231 concentration showed high performance for the diagnosis of AD dementia (AUC = 0.94 (95% CI = 0.87–1.0), Fig. 1a, b). The addition of age, sex, and *APOE* ε4 genotype in the models did not significantly increase the diagnostic performance of plasma p-tau231.

In the TRIAD cohort, plasma p-tau231 was significantly increased in AD dementia and MCI Aβ-positive groups compared to Aβ-negative groups (*P* < 0.0001). Furthermore, plasma p-tau231 was significantly increased in CU elderly Aβ-positive and MCI Aβ-positive compared to Aβ-negative groups (*P* < 0.0001, Fig. 1c, d;). The lower accuracy in distinguishing AD dementia from CU elderly Aβ-positive (AUC = 0.67) and MCI Aβ-positive (AUC = 0.72) compared with plasma p-tau181 (supplementary table 5, online resource) illustrated that plasma p-tau231 has already increased at the preclinical phase of the disease. Plasma p-tau231 was highly accurate in detecting AD in all dementia cases (AUC = 0.93). In comparison to plasma p-tau181, p-tau231 showed no significant difference in the prediction of AD dementia from non-AD dementias in the TRIAD cohort (supplementary Fig. 3, online resource); this remained true for all other comparisons between AD (supplementary table 5, online resource) and MCI Aβ-positive (supplementary table 6, online resource) with other groups included in the study. However, plasma p-tau231 was significantly better than plasma p-tau181 in distinguishing CU elderly Aβ-positive from MCI Aβ-negative (*P* < 0.05, supplementary table 7, online resource), while the reverse was true in separating CU elderly Aβ-positive from AD (*P* < 0.05, supplementary table 7, online resource). In the whole group, plasma p-tau231 correlated with plasma p-tau181 (*r* = 0.654, *P* < 0.0001), which was stronger in Aβ-positive individuals (*r* = 0.611, *P* < 0.0001) than Aβ-negative individuals (*r* = 0.444, *P* < 0.0001). Plasma p-tau231 also correlated with plasma NfL (*r* = 0.414, *P* < 0.0001).

In the primary care setting, plasma p-tau231 concentration increased progressively from CU young to elderly, MCI, and AD dementia patients (*P* < 0.0001, Fig. 1e). Plasma p-tau231 discriminated AD dementia from CU young and elderly (AUC = 0.91 and AUC = 0.75, Fig. 1f) but not from MCI (AUC = 0.63) (Fig. 1f).

In the neuropathology cohort, plasma p-tau231 was confirmed as being highly accurate for discriminating between AD dementia and non-AD neurodegenerative (AUC = 0.997, 95% CI = 0.988–1.00), Fig. 1h). Again, in this analysis, plasma p-tau231 was not significantly better than plasma p-tau181 (AUC = 0.9293, 95% CI = 0.8551–1.00) despite being numerically higher in the same patients. A stepwise increase of plasma p-tau231 between Braak I–II, III–IV and V–VI was observed (*P* = 0.0004, supplementary Fig. 4a, online resource) which was less pronounced for plasma p-tau181 (*P* = 0.0125, supplementary Fig. 4b, online resource). Plasma p-tau231 had a higher accuracy in discriminating Braak I–II and V–VI (AUC = 0.99, 95% CI = 0.97–1.00) and Braak I–II and Braak III–IV (AUC = 0.75, 95% CI = 0.61–0.98) in comparison to p-tau181 (supplementary Fig. 4c, online resource). There was no noticeable difference when observing the concentration of p-tau231 in the specific diagnosis of non-AD neurodegenerative disorders for the neuropathology cohort or the validation cohort (supplementary table 9, online resource).

In all cohorts, the plasma p-tau231 levels were approximately twofold higher in AD than CU combined (threefold higher in AD than in CU elderly-Aβ-negative). Plasma p-tau231 was 2.6-fold higher in AD compared to non-AD dementias.

### Plasma p-tau231 is closely related to CSF and PET biomarkers

**Fig. 2:**
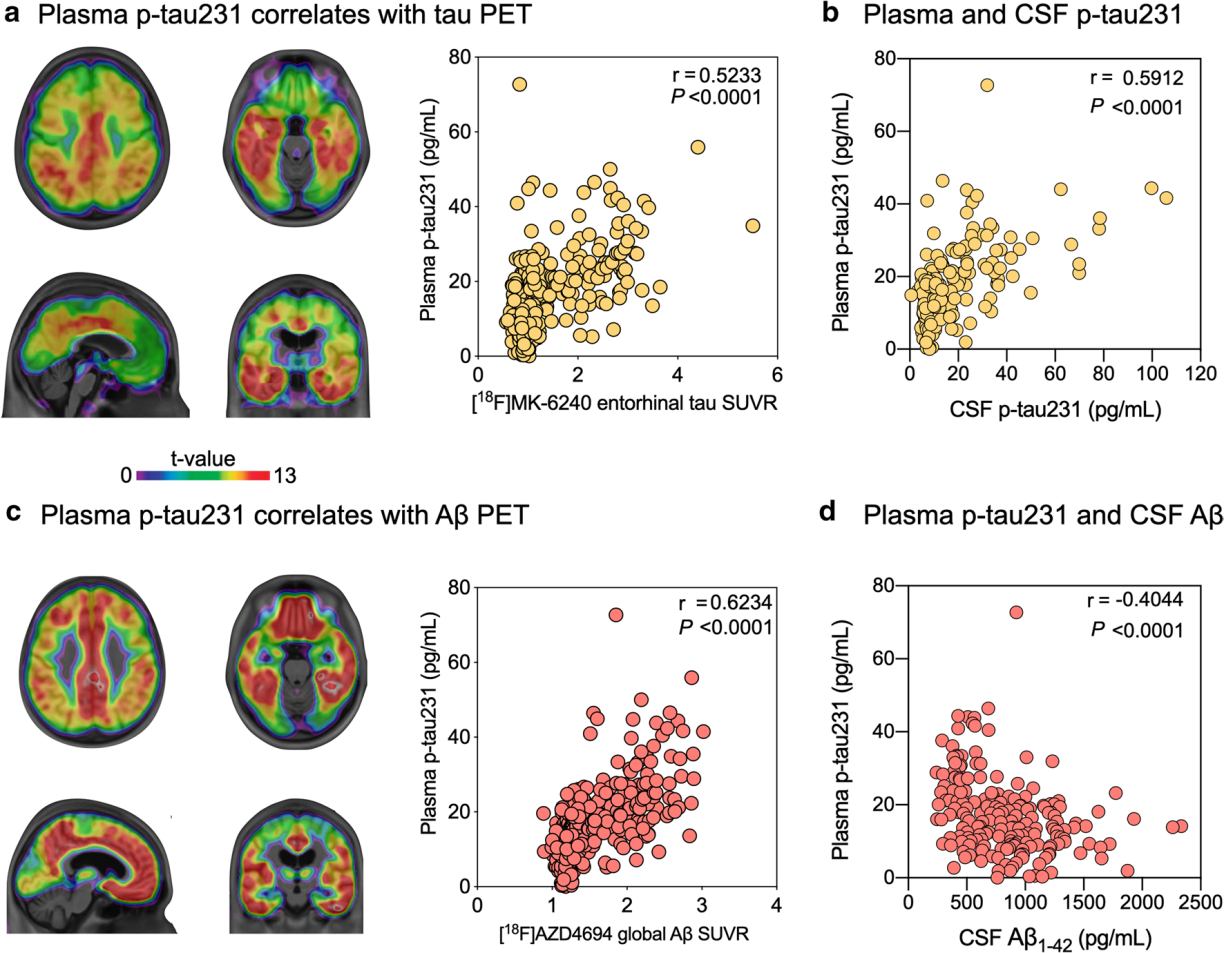
Plasma p-tau231 concentration correlates with PET and CSF tau and Aβ. The brain maps show the results of linear regressions false discovery rate corrected for multiple comparisons at *P* < 0.05, whereas the plots show the results of Spearman rank correlation between biomarkers. **a** Plasma p-tau231 concentrations were associated with [^18^F]MK-6240 SUVR across the cortex with the highest association in the temporal and posterior cingulate cortices as well as with **b** CSF p-tau231 concentrations. **c** Plasma p-tau231 correlates with [^18^F]AZD4694 SUVR across the cortex and with **d** CSF Aβ_1-42_ concentrations

In the validation cohort, plasma p-tau231 was strongly associated with tau [^18^F]MK-6240 SUVR values across the brain cortex with the highest association in the temporal and cingulate cortices as well as with CSF p-tau231 (*P* < 0.0001; Fig. 2a, b). Plasma p-tau231 also showed a strong correlation with Aβ [^18^F]AZD4694 PET across the brain cortex with the highest association in the precuneus, frontal cortex, and striatum (Fig. 2c). Interestingly, in CU individuals, the relationship between plasma p-tau231 and Aβ PET existed in both Aβ-positive (*P* < 0.01, *r* = 0.502) and Aβ-negative (*P* < 0.0001, *r* = 0.389) individuals. However, in cognitively impaired (CI) patients, plasma p-tau231 and Aβ relationship was confined to Aβ-positive individuals only (*P* < 0.001, r = 0.4*5*0). Plasma p-tau231 was also highly correlated with CSF Aβ_1-42_ (*P* < 0.0001; Fig. 2d).

### Plasma p-tau231 as an early markers of AD pathology

**Fig. 3:**
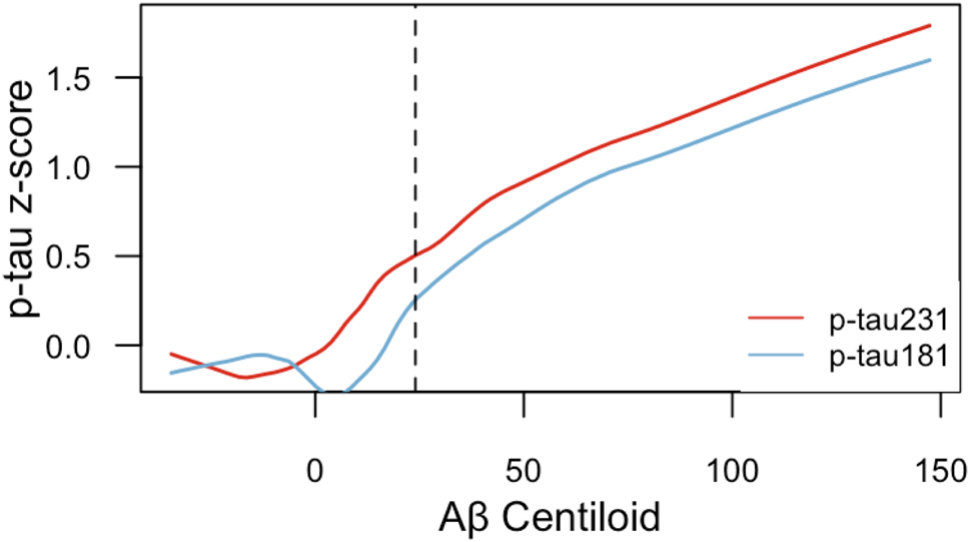
Plasma p-tau231 increases earlier than plasma p-tau181 as a function of brain Aβ deposition. The lines represent weighted regression models showing the association between *z*-scored plasma p-tau231 and p-tau181 concentrations as a function of centiloid Aβ PET levels in CU and MCI individuals (*P* < 0.0001). The vertical dashed lines represent the inflection point of the plasma p-tau231 (red), plasma p-tau181 (blue), as well as the centiloid threshold of Aβ positivity (black; centiloid = 22). The graph suggests that plasma p-tau231 raises before plasma p-tau181 with the increase of brain Aβ levels

**Fig. 4:**
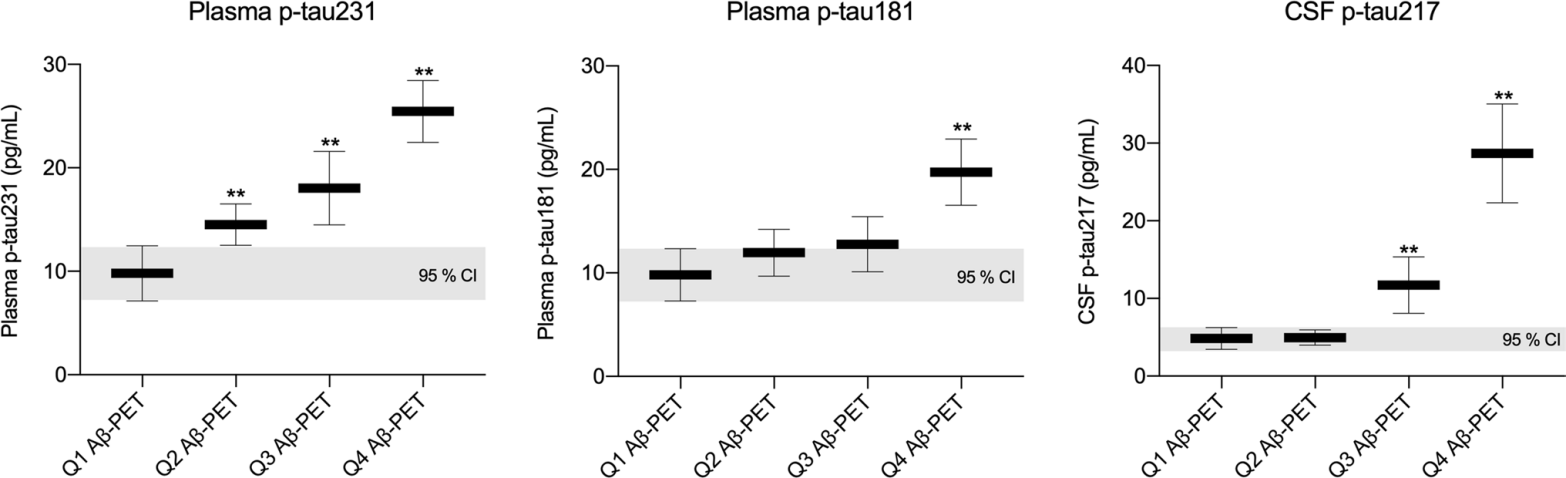
Plasma p-tau231 increases earlier that plasma p-tau181 and CSF p-tau217 as a function of Aβ PET quartiles. The horizontal grey bars shows the mean and 95% confidence intervals (CI) of plasma p-tau231 and p-tau181 as well as CSF p-tau217 in elderly individuals (CU, MCI, AD) segregated Aβ PET quartiles. Plasma p-tau231 showed abnormally increased levels from Aβ PET quartile 2 to 4; plasma p-tau181 in quartile 4, and CSF in quartiles 3–4

**Fig. 5:**
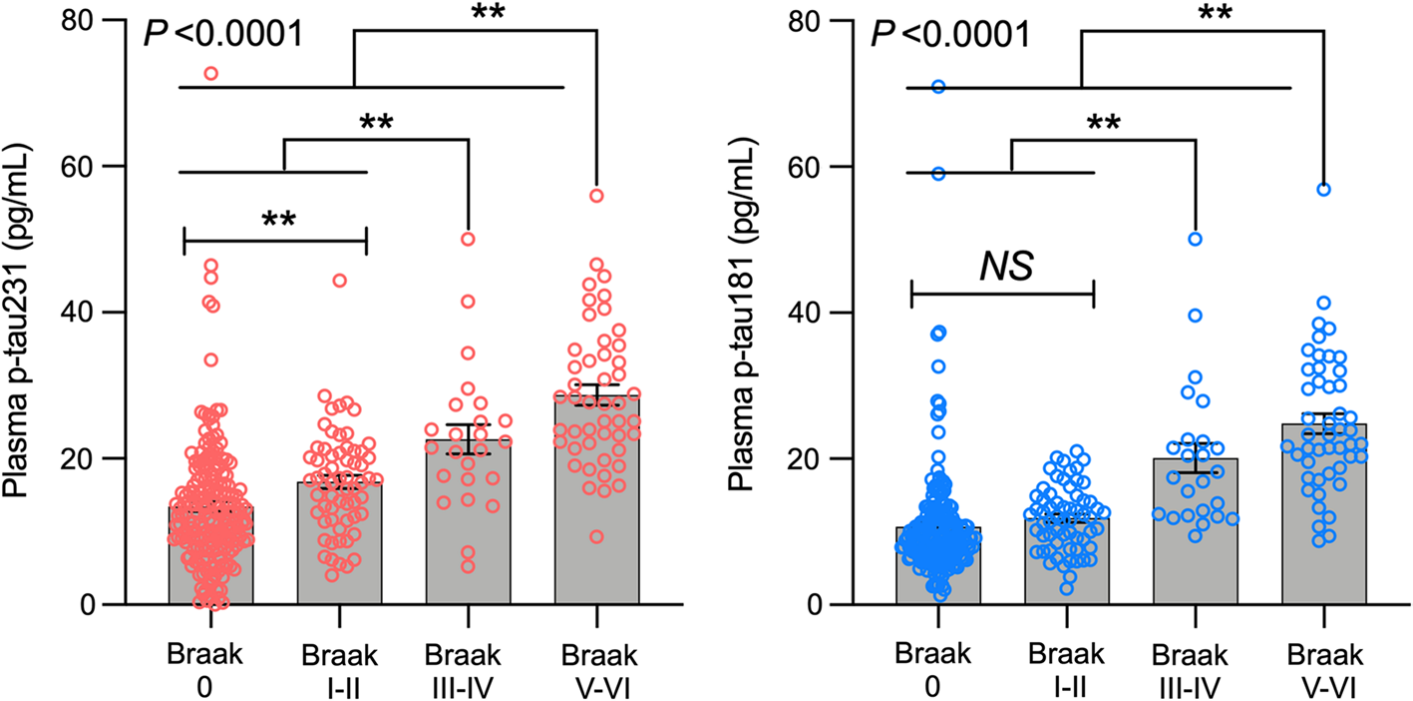
Plasma p-tau231 concentration segregates individuals across the entire Braak stage spectrum. The bars show the mean and the standard error of the mean. *P* values indicate the results of the analysis of variance models with Tukey’s post hoc multiple comparisons at ** *P* < 0.05. The figure shows that plasma p-tau231 segregates individuals across all Braak stage, whereas plasma p-tau181 did not differentiate individuals who were in Braak stage 0 from those in Braak stage I–II, suggesting plasma p-tau231 raises early in the AD pathophysiological phase

Plasma p-tau231 (AUC = 0.82 (95% CI 0.76–0.92)) differentiated CU Aβ-negative from CU Aβ-positive, and demonstrated a numerically higher AUC than plasma p-tau181 (supplementary Fig. 5, online resource). The inflection points of the weighted regression curves suggested that plasma p-tau231 demonstrates an earlier increase than plasma p-tau181 as a function of Aβ deposition load (Fig. 3) and that this was prior to the threshold of Aβ positivity had been achieved (Aβ centiloid = 22). Plasma p-tau231 showed abnormal levels from Aβ PET quartiles 2–4, whereas plasma p-tau181 and CSF p-tau217 became abnormal in quartile 4 and quartile 3, respectively (Fig. 4). Furthermore, plasma p-tau231 segregated individuals across early and late Braak stages of tau tangles accumulation (Fig. 5) including Braak I-II, which was not observed for plasma p-tau181.

### Plasma p-tau231 predicts longitudinal AD progression

A subsample of individuals in the TRIAD cohort (*n* = 126) had a 1-year follow-up structural MRI and cognitive assessment. Plasma p-tau231 predicted 1-year worsening in MMSE scores and hippocampal atrophy (Table 3).

**Table 3:** Plasma p-tau231 concentration predicts longitudinal changes in hippocampus volume and cognitive decline

ΔHV ~ P-tau231 + Age + Education + Sex + Aβ				ΔMMSE ~ P-tau231 + Age + Education + Sex + Aβ			
	Beta	Std. error	*p* value		Beta	Std. error	*p* value
P-tau231	− 0.0023	0.0011	0.0321*	P-tau231	− 0.0424	0.0178	0.0196*
Age	− 0.0003	0.0010	0.7340	Age	0.0310	0.0289	0.2866
Education	0.0038	0.0028	0.1835	Education	− 0.0658	0.0502	0.1935
Sex	0.0281	0.0208	0.1780	Sex	− 0.2403	0.3789	0.5275
Aβ	− 0.0005	0.0034	0.8886	Aβ	− 0.0244	0.0616	0.6934

**ΔHV ~ P-tau181 + Age + Education + Sex + Aβ**				**ΔMMSE ~ P-tau181 + Age + Education + Sex + Aβ**			
	Beta	Std. error	*p* value		Beta	Std. error	*p* value
P-tau181	− 0.0025	0.0012	0.0452*	P− tau181	− 0.0751	0.0216	0.0008*
Age	− 0.0005	0.0010	0.6335	Age	0.0259	0.0278	0.3542
Education	0.0036	0.0029	0.2151	Education	− 0.0657	0.0487	0.1808
Sex	0.0307	0.0207	0.1398	Sex	− 0.1762	0.3643	0.6298
Aβ	− 0.0002	0.0034	0.9618	Aβ	− 0.0106	0.0594	0.8588

The table show the results of beta values, standard error, and p values coefficients of linear regression models. Both plasma p− tau231 and p− tau181 precicted with 1− year decrease in hippocampal volume (HV) (neurodegeneration) and 1− year worsening in MMSE scores (cognitive decline) in a subset of TRIAD individuals (n = 126), accounting for years of age, years of formal education, sex (female = 1), and plasma Aβ levels. For changes (Δ) in MMSE score and HV, lower scores represent a cognitive decline and decrease in brain volume, respectively

## Discussion

In this study, we report the first ultrasensitive immunoassay for the accurate quantification of phosphorylated tau at threonine 231 in plasma and investigated its potential in multiple scenarios across the AD continuum, primary care utility and against neuropathological confirmation. The findings show that plasma p-tau231, like plasma p-tau181, demonstrates high diagnostic accuracy in detecting AD in the dementia stage of the disease, which included neuropathologically confirmed cases of AD and non-AD neurodegenerative diseases. Plasma p-tau231 also discriminated Aβ-positive CU and MCI cases from Aβ-negative CU elderly with high accuracy and correlated strongly with Aβ and tau PET. The novel findings of plasma p-tau231 is that it was shown to (i) increase and correlate with Aβ PET, prior to formal Aβ PET positivity, (ii) significantly increases earlier than plasma p-tau181 in CU individuals, (iii) segregates Aβ PET quartiles better than plasma p-tau181 and CSF p-tau217 and iv) detects early tau deposition, which was not observed for plasma p-tau181.

The high specificity of CSF p-tau for AD is an established finding [12, 21, 26, 46]. This conclusion is now an ever-increasing outcome in blood [10, 29, 34, 48] and makes plasma p-tau measures the main candidate for an AD-specific blood biomarker. To date, no report of plasma p-tau231 has been described in blood, despite it being a widely reported CSF biomarker [2, 11, 14, 15, 18, 20, 30, 33, 53, 58]. The ultrasensitive Simoa assay presented in this study measures specific tau entities that include both the N-terminal part of the molecule and a phosphorylated epitope including amino acid 231 (N-p-tau231, supplementary Fig. 1–2, online resource), as verified by mass spectrometry and immunoprecipitation experiments. Importantly, the strong correlation of plasma with CSF p-tau231 supports the notion that this assay specifically measures a brain-derived phospho-form of tau. Furthermore, as a proof-of-principle, our plasma p-tau231 assay has similar diagnostic performances as the previously described plasma p-tau181 assay [29] replicating the multitude of studies demonstrating that CSF p-tau231 and CSF p-tau181 have identical performances in the clinical stage of AD dementia. Specifically, plasma p-tau231 had high diagnostic accuracy in distinguishing AD from non-AD dementias (AUC = 0.93), which included primary tauopathies (e.g.*,* frontotemporal dementia, progressive supranuclear palsy and corticobasal degeneration) and this is most likely the main clinical application of plasma p-tau. This high discrimination between AD and non-AD was verified in patients with neuropathologically confirmed post-mortem tissue (AUC = 0.99) and despite all individuals been given a clinical diagnosis of probable AD during life—confirming the potential clinical utility of plasma p-tau. Similarly, plasma p-tau231 could also discriminate AD from MCI cases without underlying AD pathology (AUC = 0.89), which is also a potential challenge observed in clinical settings. Plasma p-tau231 also separated AD from Aβ-negative CU elderly (AUC = 0.92) and young adults (AUC = 0.95) with very high accuracy. However, it demonstrated, much like the CSF comparisons that have preceded this study, that plasma p-tau231 was not significantly superior nor inferior to plasma p-tau181 in the aforementioned analysis. One exception to this being plasma p-tau231 being able to distinguish CU Aβ-positive participants from Aβ-negative MCI cases with higher accuracy and significantly superior to plasma p-tau181 (supplementary table 7, online resource).

As the performance of plasma p-tau231 was shown to be equivalent to plasma p-tau181 in several domains (*e.g.,* distinguishing AD and non-AD and identifying Aβ positivity), and combining these biomarkers did not show significant improvement in their performance in the cases tested in this study—what does a measure of plasma p-tau231 offer compared to other plasma p-tau biomarkers that have been previously described? Recently, we performed two independent studies comparing CSF p-tau biomarkers (p-tau181, p-tau217, p-tau231) in the AD continuum. Suarez-Calvet et al*.* [58] demonstrated, in a CU preclinical cohort, that CSF p-tau231 was increased more prominently with emerging pathology, whether Aβ pathophysiology was measured in CSF or using PET. In support of this, CSF p-tau231 had a markedly stronger relationship with increasing Aβ in individuals prior to the threshold of Aβ positivity [3]. These associations were localized to focal Aβ retention in brain regions corresponding to regions involved in the default mode network, a previously reported site of early Aβ pathology accumulation [49]. Neuropathological findings also highlight tau phosphorylation at threonine 231 to be a key marker of early disease as it is a predominate feature of pre-neurofibrillary tangle pathology [19] and one of the earliest events in the cascade of phosphorylation that modulates tubulin assembly [1, 55]. Together, these recent CSF findings and neuropathological evidence paired with the successful replication of p-tau biomarkers from *CSF-to-blood*, we hypothesized that plasma p-tau231 would indeed mirror a very early increase in the AD continuum. We observed a numerically higher AUC of plasma p-tau231 as compared to p-tau181 in the prediction of Aβ positivity in CU individuals, which mirrors CSF findings [58]. However, no significant differences between plasma p-tau231 and p-tau181 were found in this analysis, likely attributed to the finding that soluble p-tau231 is already increasing in relation to Aβ pathology prior to Aβ PET positivity. We demonstrated this by showing that the inflection point of p-tau231, and consequent increase, occurs considerably earlier than plasma p-tau181 as a function of brain Aβ deposition in dementia-free participants—this change was substantially earlier than the threshold of centiloid Aβ PET positivity. Moreover, plasma p-tau231 shows a progressive stepwise increase with Aβ levels in Aβ-negative individuals which was not observed for plasma p-tau181 and even CSF p-tau217.

The early and pronounced increase of plasma p-tau231, as shown in this study, has obvious implications for therapeutic trials, which are increasingly seeking to recruit individuals in the earliest stages of AD pathophysiology. Aβ deposits are a relatively late consequence of Aβ aggregation in AD—thus the preclinical phase of AD (Aβ deposition is present with no clinical symptoms) is the desired population target of many antibody-mediated clearances of Aβ. Plasma biomarkers have been proposed to act as a prescreening tool to detect Aβ-positive individuals. In our recent analysis on the ADNI cohort, we described the substantial cost-savings that a robust plasma biomarker (e.g., plasma p-tau181) could have if introduced into the recruiting process of CU individuals with Aβ pathology [27]. The present study suggests that plasma p-tau231 may be superior at this task (AUC_p-tau231_ = 0.83 *versus* AUC = 0.77_p-tau181_ in this study or AUC_p-tau181_ = 0.70 as reported by Karikari et al. [27]). However, recent in vitro evidence demonstrates that pre-clinical AD may appear as a consequence of a much earlier pathogenic events, prior any in vivo detection of Aβ aggregation, and by reducing the concentration of Aβ during a “*pre-amyloid*” phase halts Aβ plaque formation and AD onset [63]. Therefore, the pre-clinical phase of AD maybe a too advanced state in the disease course for the therapeutic agent to optimally target the most pathogenic phase of the disease. We have shown that plasma p-tau231 can stage Aβ-negative individuals based on their proximity to the threshold for Aβ-positivity. Therefore, a critical use of plasma p-tau231 would be a pre-screen to identify participants who have higher levels of Aβ burden but who have not reached the threshold of positivity by PET (quartile 2 or 3, in our study)—namely, the “*pre-amyloid*” phase. Our analysis suggests that only plasma p-tau231 can identify both quartile 2 and quartile 3 from quartile 1, whereas plasma p-tau181 cannot distinguish between quartiles 1–3 and CSF p-tau217 (inferring on plasma p-tau217) can identify quartiles 3 and 4 but not quartile 2. This distinctly shows the early increases of plasma p-tau231 and the potential to rapidly highlight a risk group, an intermediate between Aβ-negative and Aβ-positive, which would be attractive and more beneficial group to target with anti-Aβ therapeutics. Further, a negative plasma p-tau231 test (*e.g.,* quartile 1, Fig. 4) not only indicates no clinical AD (and Aβ-negativity) but also suggests no emerging pathology is present—thus, would satisfy as definitive confirmation of controls for a trial design, interventions and research cohorts. At the very least, individuals highlighted as quartile 3 by plasma p-tau231 would not be adequate as definitive Aβ-negative controls.

In light of the marginal clinical benefit of the clinical trials with Aβ-targeting drugs [61] and findings that tau pathology is more related to the clinical presentation of AD—tau therapies are emerging as possible frontrunners in the search for an effective treatment for AD [24]. Nevertheless, common issues with anti-Aβ trial recruitment are likely to occur in the anti-tau trials as well, *e.g.*, methods to identify underlying pathology are too costly and invasive. Plasma p-tau231 strongly associated with tau PET demonstrating its relationship with insoluble tau deposits. Plasma p-tau231 was associated with [^18^F]MK-6240 retention in all tested regions but was strongest in the entorhinal cortex (Braak I–II) which is one of the earliest regions affected in AD and a site of early tau deposition. When employing the Braak staging system by tau PET, plasma p-tau231 segregated individuals across early and late Braak stages of tau tangle accumulation in a stepwise manner. This was also observed in the neuropathology cohort, despite being a small number of subjects in the Braak I–II group. Importantly, in the validation cohort, a significant increase of plasma p-tau231 was observed in Braak I–II as compared to Braak 0 (tau PET-negative), which was not observed for plasma p-tau181. This earlier increase has previously been described as an important feature of CSF p-tau231 [16], which is now replicated in blood. The non-significant difference observed for plasma p-tau181 at early Braak stages (Braak 0 *versus* Braak I-II) has been verified by other groups [25, 60]. This demonstrates that plasma p-tau231 could act as an early marker of aberrant tau phosphorylation but also insoluble tau deposition, which can be utilised in future tau interventional studies. The biomarker could also be used in genetic and epidemiological studies to find novel risk and resilience factors specific for AD-type neuropathology, and validate whether those associated with dementia are specifically related to AD.

Tau in CSF and plasma is predominantly N-terminal to mid-region truncated fragments [7, 54]. Furthermore, biochemical and mass spectrometry methods show that the phosphorylation profile of soluble tau in AD brain was highly correlated to that in AD CSF [22]. Thus, future longitudinal studies in specific stages of AD may reveal a specific role for specific phospho-tau forms in the evolution of that stage *e.g.,* p-tau231 and p-tau217 being early markers linking Aβ and tau pathology before tau PET becomes detectable, while in the AD dementia stage, decreases in tau phosphorylated at serine 202 and p-tau231 may reflect the ‘transition’ of soluble tau into insoluble tau in AD brain [22].

A limitation of this study is that we cannot directly compare plasma p-tau231 with plasma p-tau217. However, using CSF p-tau217 as a substitute for plasma p-tau217 (which is very likely a stronger predictor of disease pathophysiology) we are confident in our conclusions. Although our findings show that plasma p-tau231 assay can identify AD in a primary care setting, the primary care cohort had no confirmatory biomarkers, of individuals with MCI and identification of preclinical AD in CU adults. Given the early increases in p-tau231, these assessments would have reduced the overlap in plasma p-tau231 concentrations between CU, MCI and AD. Furthermore, the use of cross‐sectional Aβ PET Centiloids as a proxy of time in the disease was employed and it is not guaranteed that a greater Aβ PET SUVR is indicative of more advanced disease state. Lastly, to definitively and fully describe the ordinal sequence of plasma p-tau biomarkers, from preclinical to symptomatic phases of the disease, large-scale longitudinal studies with multiple time points are required.

To conclude, our novel plasma p-tau231 assay is the first description of this tau phosphorylation site in the blood which identifies AD pathology, at the dementia stage, to the same degree as plasma p-tau181. However, plasma p-tau231 has additional advantages; (1) begins to increase with subtle Aβ deposition, prior to Aβ PET threshold has been attained, and noticeably before p-tau181 and segregates Aβ PET better than p-tau217; (2) increases in p-tau231 with early NFT deposition in the entorhinal cortex (Braak 0 *versus* Braak I–II) which not observed for p-tau181. Thus, plasma p-tau231 demonstrates excellent clinical utility as a rapid screening test for AD but may serve as a superior staging biomarker of emerging AD pathology allowing clinical trials to targeting vulnerable populations under the threshold of Aβ positivity and early tau deposition.

## Supplementary Information

Below is the link to the electronic supplementary material.

Supplementary file1 (DOCX 1131 KB)
