# Supplementary material for: Plasma p-tau231: a new biomarker for incipient Alzheimer’s disease pathology
Source: Acta Neuropathol. 2021 Feb 14;141(5):709–24. doi: 10.1007/s00401-021-02275-6 (PMC8043944; doi:10.1007/s00401-021-02275-6)
Supplement: Supplementary file 1 — Supplementary file1 (DOCX 1131 KB) [file 401_2021_2275_MOESM1_ESM.docx]

**Supplementary Data**

**Plasma p-tau231: a new biomarker for incipient Alzheimer’s disease pathology**

Nicholas J. Ashton ^1, 2, 3, 4*^, Tharick A. Pascoal ^5, 6*^, Thomas K. Karikari ^1^, Andréa L. Benedet ^1, 5^, Juan Lantero-Rodriguez ^1^, Gunnar Brinkmalm ^1^, Anniina Snellman ^1^, Michael Schöll ^1, 2, 10^, Claire Troakes ^14^, Abdul Hye ^3, 4^, Serge Gauthier ^7^, Eugeen Vanmechelen ^8^, Henrik Zetterberg ^1, 9, 10, 11^, Pedro Rosa Neto ^1, 12, 13^**^†^**, Kaj Blennow ^1, 9^**^†^**

^1^Department of Psychiatry and Neurochemistry, Institute of Neuroscience & Physiology, the Sahlgrenska Academy at the University of Gothenburg, Mölndal, Sweden; ^2^Wallenberg Centre for Molecular and Translational Medicine, University of Gothenburg, Gothenburg, Sweden; ^3^King’s College London, Institute of Psychiatry, Psychology and Neuroscience, Maurice Wohl Institute Clinical Neuroscience Institute, London, UK. ^4^NIHR Biomedical Research Centre for Mental Health and Biomedical Research Unit for Dementia at South London and Maudsley NHS Foundation, London, UK; ^5^Translational Neuroimaging Laboratory, McGill Centre for Studies in Aging, McGill University, Montreal, QC, Canada; ^6^Department of Psychiatry and Neurology, University of Pittsburgh, Pittsburgh, PA, USA. ^7^Alzheimer’s Disease Research Unit, The McGill University Research Centre for Studies in Aging, Montreal, McGill University, Montreal, QC, Canada; ^8^ADx NeuroSciences, Technologiepark 94, 9052 Ghent, Belgium; ^9^Clinical Neurochemistry Laboratory, Sahlgrenska University Hospital, Mölndal, Sweden; ^10^Department of Neurodegenerative Disease, UCL Institute of Neurology, Queen Square, London, UK; ^11^UK Dementia Research Institute at UCL, London, UK; ^12^Montreal Neurological Institute, Montreal, QC, Canada; ^13^Department of Neurology and Neurosurgery, McGill University, Montreal, QC, Canada

^14^Department of Basic and Clinical Neuroscience, Institute of Psychiatry, Psychology & Neuroscience, King’s College London, London, UK

Corresponding authors:

Dr. Nicholas J. Ashton, Institute of Neuroscience and Physiology, Department of Psychiatry and Neurochemistry, Institute of Neuroscience and Physiology, The Sahlgrenska Academy, University of Gothenburg, Mölndal, Sweden

Tel.: +44 (0) 7380268766

Email: [nicholas.ashton@gu.se](mailto:nicholas.ashton@gu.se)

Kaj Blennow, MD, PhD, Institute of Neuroscience and Physiology, Department of Psychiatry and Neurochemistry, University of Gothenburg, Mölndal, Sweden

Tel: +46 031-342 10 00

Email: [kaj.blennow@neuro.gu.se](mailto:kaj.blennow@neuro.gu.se)

^*^Authors contributed equally

**^†^**Senior author contributed equally

**CONTENTS**

**SUPPLEMENTARY METHODS**

Analytical validation of p-tau231 assay

**SUPPLEMENTARY TABLES**

Supplementary Table 1. Dilution linearity of plasma p-tau231 assay.

Supplementary Table 2. Spike recovery performance of the p-tau231 assay

Supplementary Table 3. Analytical performance of the p-tau231 assay

Supplementary Table 4. Characteristics of the discovery cohort

Supplementary Table 5. Area under the curve comparisons of AD from other groups

Supplementary Table 6. Area under the curve comparisons of MCI Aβ+ from other groups

Supplementary Table 7. Area under the curve comparisons of elderly CU Aβ+ from other groups

Supplementary Table 8. Area under the curve comparisons of elderly CU Aβ– from other groups

**SUPPLEMENTARY FIGURES**

Supplementary Figure 1. Specificity of p-tau231 assay.

Supplementary Figure 2. Validation of the specificity of the p-tau231 and p-tau181 assays by immunoprecipitation-mass spectrometry

Supplementary Figure 3. Plasma p-tau231 differentiates individuals with non-AD and AD dementia.

Supplementary Figure 4. Plasma p-tau231 has a stepwise increase in Braak stages in neuropathologically confirmed cases.

Supplementary Figure 5. Plasma p-tau231 differentiate individuals CU cognitive Aβ positive from Aβ negative.

**SUPPLEMENTARY METHODS**

**Analytical validation of p-tau231 assay**

The assay validation focused on dilution linearity, spike recovery and antibody specificity. For dilution linearity (Table S1), plasma samples were analyzed undiluted or diluted (2-fold, 4-fold and 8-fold).

The % recovery was calculated using the following equation:

% recovery at dilution x = (Concentration at dilution x) / (Concentration of undiluted sample) ×100

For spike recovery (Table S2), neat plasma samples as well as the assay diluent only were each analyzed untreated (non-spiked) or ‘spiked’ with low or high concentrations of recombinant tau phosphorylated at threonine-231.

The following equation was used to calculate spike recovery:

% recovery = (Concentration of spiked sample) / (Concentration of non-spiked + concentration of spiked buffer) ×100

To demonstrate that the assay is specific to p-tau231, identical CSF samples were divided into two aliquots (aliquot 1 = untreated; aliquot 2 = immunodepleted with the p-tau231 antibody). The concentrations of p-tau231 and p-tau181 in the depleted and untreated pairs of samples were then measured using the p-tau231 assay (Fig. S1). The CSF p-tau231 was quantified by the same Simoa assay as plasma.

**SUPPLEMENTARY TABLES**

**Supplementary Table 1. Dilution linearity of plasma p-tau231 assay.**

| Sample No. | Fold Dilution | Calc. conc. (pg/mL) | Conc. CV (%) | Mean Calc. conc. (pg/mL) | Dilution corrected conc. (pg/mL) | % Recovery |
| --- | --- | --- | --- | --- | --- | --- |
| 1 | 1 | 19.1  23.3 | 14.1 | 20.7 | 20.7 |  |
|  | 2 | 8.7  8.7 | 0.0 | 8.7 | 17.4 | 84.1 |
|  | 4 | 5.1  5.7 | 7.8 | 5.4 | 21.6 | 104.3 |
|  | 8 | 2.2  2.7 | 14.5 | 2.25 | 18 | 87.5 |
| 2 | 1 | 18.2  17.8 | 1.6 | 18 | 18 |  |
|  | 2 | 9.8  9.9 | 0.7 | 9.9 | 19.8 | 110.2 |
|  | 4 | 5.2  4.8 | 5.7 | 5 | 20 | 111.2 |
|  | 8 | 2.5  2.5 | 0.0 | 2.5 | 20 | 111.2 |

**Supplementary Table 2. Spike recovery performance of the p-tau231 assay**

| **Sample** | **Treatment** | **Observed conc. (pg/ml)** | | **%CV conc.** | **Expected conc. (pg/ml)** | **% recovery** |
| --- | --- | --- | --- | --- | --- | --- |
|  |  | **Measured** | **Mean** |  |  |  |
| **Plasma 1** | Neat | 14.66 | 14.80 | 1.40 |  |  |
|  |  | 14.95 |  |  |  |  |
|  | + high spike | 33.57 | 34.25 | 2.79 | 34.80 | 98.4 |
|  |  | 34.92 |  |  |  |  |
|  | + low spike | 17.12 | 17.31 | 1.54 | 18.80 | 92.1 |
|  |  | 17.50 |  |  |  |  |
| **Plasma 2** | Neat | 13.85 | 14.10 | 2.44 |  |  |
|  |  | 14.34 |  |  |  |  |
|  | + high spike | 32.42 | 32.75 | 1.45 | 34.10 | 96.0 |
|  |  | 33.09 |  |  |  |  |
|  | + low spike | 16.15 | 16.31 | 1.42 | 18.10 | 90.1 |
|  |  | 16.47 |  |  |  |  |
| **Buffer control** | Buffer + high spike | 20.00 | 18.66 | 10.19 | 20.00 | 106.9 |
|  |  | 17.31 |  |  |  |  |
|  | Buffer + low spike | 3.58 | 3.42 | 6.39 | 4.00 | 85.5 |
|  |  | 3.27 |  |  |  |  |

**Supplementary Table 3. Analytical performance of the p-tau231 assay**

|  | **Study cohort** | | | |
| --- | --- | --- | --- | --- |
|  | **Discovery** | **Validation** | **Primary care** | **Neuropathology** |
|  | % CV (SD) | % CV (SD) | % CV (SD) | % CV (SD) |
| Low-iQC  5.5 pg/mL | 11.7% (0.9) | 15.2% (0.7) | 13.0% (1.1) | 10.5% (0.9) |
| Medium-iQC  23.8 pg/mL | 6.7% (1.8) | 10.1% (2.0) | 7.7% (1.6) | 12% (2.1) |
| High-iQC  32.1 pg/mL | 8.2% (2.0) | 6.2% (1.5) | 4.2% (1.0) | 9.2% (1.2) |

**Supplementary Table 4. Characteristics of the discovery cohort**

|  | **Discovery cohort (n=38)** | |
| --- | --- | --- |
|  | **CU older adults (n=18)** | **AD**  **(n=20)** |
| Age, years | 63.9 (11.1) | 72.2 (5.1) ^*^ |
| Sex  Men  Women | 13 (72%)  5 (28%) | 10 (50%)  10 (50%) |
| *APOE* ε4 carriership | **…** | **…** |
| Education, years | **…** | **…** |
| CSF biomarkers    Aβ42  P-tau181  T-tau | 840.2 (175.9)  35.9 (10.1)  228.4 (82.3**)** | 388.2 (70.5) ^*^  89.8 (29.8) ^*^  681.2 (233.9) ^*^ |

*p<0·05 compared with cognitively unimpaired older adults.

**Supplementary Table 5. Area under the curve comparisons of AD from other groups**

| Plasma Biomarker | **AD versus** | | | | | |
| --- | --- | --- | --- | --- | --- | --- |
|  | Young adults | Elderly Aβ-negative | Elderly Aβ-positive | MCI Aβ-negative | MCI Aβ-positive | Non-AD |
| P-tau231 | 0.95  (0.91–0.99) | 0.92^*^  (0.86–0.97) | 0.67  (0.55–0.81) | 0.88^*^  (0.81–1.00) | 0.72  (0.61–0.83) | 0.93^*^  (0.88–0.99) |
| P-tau181 | 0.97  (0.94–1.00) | 0.94^*^  (0.90–0.96) | 0.79  (0.660–0.91) | 0.86^*^  (0.70–1.00) | 0.76  (0.66–0.87) | 0.94^*^  0.89–0.99) |
| NfL | 0.99 (0.98–1.00) | 0.80 (0.76–0.86) | 0.66 (0.54–0.70) | 0.70 (0.62–0.72) | 0.73 (0.70–0.76) | 0.57 (0.50–0.61) |

*p<0·05 compared with NfL

**Supplementary Table 6. Area under the curve comparisons of MCI Aβ+ from other groups**

| Plasma Biomarker | **MCI Aβ-positive versus** | | | | | |
| --- | --- | --- | --- | --- | --- | --- |
|  | Young adults | Elderly Aβ-negative | Elderly Aβ-positive | MCI Aβ-negative | AD | Non-AD |
| P-tau231 | 0.88  (0.81–0.96) | 0.80^*^  (0.72–0.88) | 0.52  (0.38–0.66) | 0.80^*^  (0.64–0.96) | 0.71  (0.61–0.83) | 0.83^*^  (0.74–0.93) |
| P-tau181 | 0.89  (0.82–0.97) | 0.83^*^  (0.76–0.90) | 0.60  (0.46–0.74) | 0.75^*^  (0.57–0.94) | 0.76  (0.66–0.87) | 0.84^*^  (0.74–0.94) |
| NfL | 0.98† (0.96–1.00) | 0.55 (0.45–0.66) | 0.59 (0.46–0.73) | 0.50 (0.27–0.72) | 0.74 (0.62–0.85) | 0.53 (0.31–0.75) |

*p<0·05 compared with NfL.

†p<0·05 compared with p-tau231 and p-tau181

**Supplementary Table 7. Area under the curve comparisons of elderly CU Aβ+ from other groups**

| Plasma Biomarker | **Elderly CU Aβ-positive versus** | | | | | |
| --- | --- | --- | --- | --- | --- | --- |
|  | Young adults | Elderly Aβ-negative | MCI Aβ-negative | MCI Aβ-positive | AD | Non-AD |
| P-tau231 | 0.90  (0.82–0.98) | 0.83^*^  (0.74–0.91) | 0.83^*‡^  (0.67–0.96) | 0.52  (0.38–0.66) | 0.67  (0.55–0.81) | 0.86^*^  (0.77–0.96) |
| P-tau181 | 0.85  (0.75–0.95) | 0.77  (0.67–0.86) | 0.70  (0.50–0.90) | 0.60  (0.46–0.74) | 0.79^*^  (0.66–0.91) | 0.74^*^  (0.60–0.88) |
| NfL | 0.99†  (0.98–1.00) | 0.67  (0.56–0.78) | 0.60  (0.36–0.82) | 0.59  (0.46–0.73) | 0.66  (0.54–0.70) | 0.56  (0.33–0.79) |

*p<0·05 compared with NfL.

†p<0·05 compared with p-tau231 and p-tau181. ‡ p<0·05 compared with p-tau181.

**Supplementary Table 8. Area under the curve comparisons of elderly CU Aβ– from other groups**

| Plasma Biomarker | **Elderly CU Aβ-negative versus** | | | | | |
| --- | --- | --- | --- | --- | --- | --- |
|  | Young adults | Elderly Aβ positive | MCI Aβ negative | MCI Aβ positive | AD | Non-AD |
| P-tau231 | 0.67  (0.56–0.78) | 0.83^*^  (0.74–0.91) | 0.56  (0.35–0.78) | 0.80^*^  (0.72–0.88) | 0.93^*^  (0.88–0.99) | 0.59  (0.47–0.71 |
| P-tau181 | 0.63  (0.53–0.74) | 0.77  (0.67–0.86) | 0.59  (0.41–0.77) | 0.83^*^  (0.76–0.90) | 0.94^*^  (0.89–0.99) | 0.51  (0.38–0.64) |
| NfL | 0.97†  (0.95–0.99) | 0.67  (0.56–0.78) | 0.55  (0.32–0.78) | 0.55  (0.45–0.66) | 0.80  (0.76–0.86) | 0.58  (0.37–0.79) |

*p<0·05 compared with NfL.

†p<0·05 compared with p-tau231 and p-tau181.

**Supplementary Table 9. P-tau231 levels in non-AD neurodegenerative disorders**

| **Non-AD neurodegenerative disorder** | **TRIAD cohort** | | **Neuropathology cohort** | |
| --- | --- | --- | --- | --- |
|  | *n* | p-tau231, pg/mL, mean (SD) | *n* | p-tau231, pg/mL, mean (SD) |
| Frontotemporal Dementia | 10 | 10.87 (2.88) | 3 | 13.04 (3.5) |
| Progressive Supranuclear Palsy | 2 | 16.12 (2.44) | 3 | 13.34 (1.26) |
| Cortical Basal Degeneration | 1 | 10.92 | NA | NA |
| Hippocampal Sclerosis | 1 | 1.98 | NA | NA |
| Primary Progressive Aphasia | 1 | 11,47 | NA | NA |
| Vascular Cognitive Impairment | 10 | 12.40 (3.1) | 2 | 11.69 (4.0) |
| Cerebral Amyloid Angiopathy | 1 | 10.03 | 1 | 9.4 |
| Lewy Body Dementia | NA | NA | 2 | 15.6 (4.51) |
| Mild Cognitive Impairment (Aβ negative) | 11 | 13.23 (5.67) | NA | NA |

**SUPPLEMENTARY FIGURES**

**
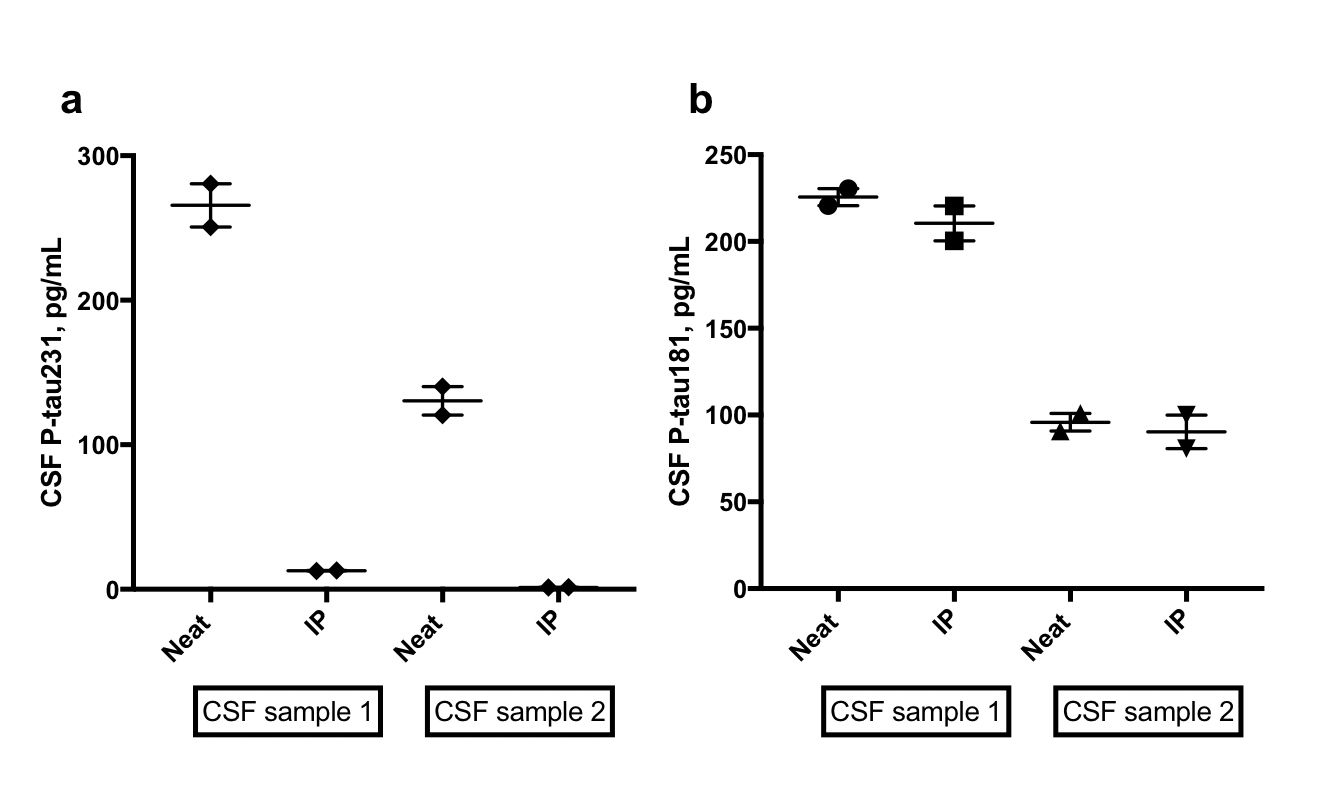
**

**Supplementary Figure 1. Specificity of p-tau231 assay.** Aliquots from two different CSF samples were analysed untreated (neat) or immunodepleted with the capture. It was shown that >99% of the signal was lost for p-tau231 after immunodepletion (a) whereas only when 5–10% of p-tau181 signal was lost (b) in the same samples, indicating that removing p-tau231 signal does not significantly affect p-tau181 levels.


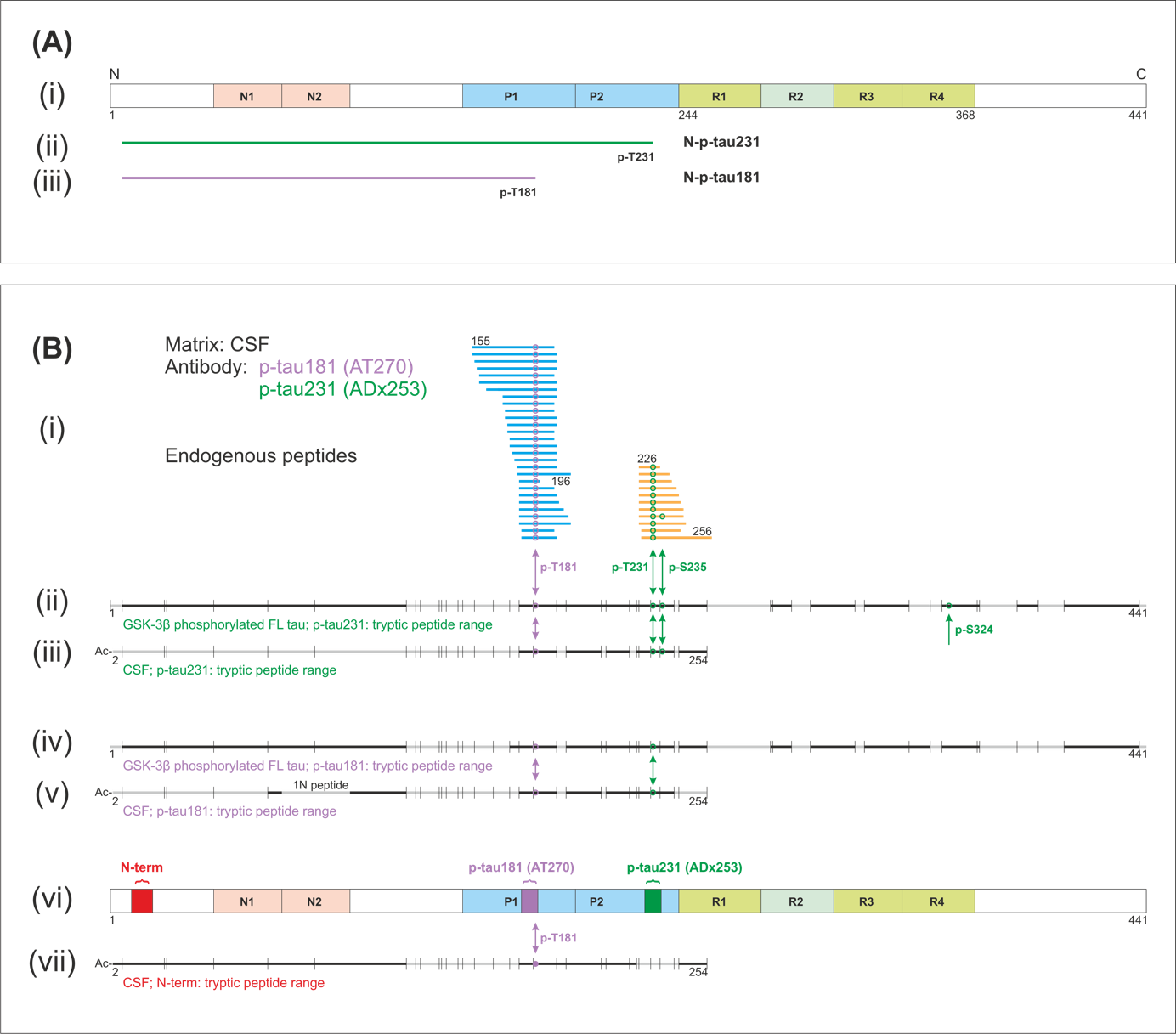


**Supplementary Figure 2. Validation of the specificity of the p-tau231 and p-tau181 assays by immunoprecipitation-mass spectrometry.** (a) Schematic of full-length tau-441 with the different regions (i) and the tau peptide fragments detected using p-tau231 (ii) and p-tau181 (iii), respectively. (b) Endogenous and tryptic peptides captured with the respective antibodies and identified by MS. Using p-tau231 11 endogenous peptides spanning aa 226-256, all phosphorylated at T231, including one peptide that was doubly phosphorylated at T231 and T235, were detected in CSF (i). In (ii), the range of tryptic peptides identified from glycogen synthase kinase (GSK)-3β-phosphorylated full-length tau-441 (the assay calibrator; SignalChem #TO8-50FN), immunoprecipitated using the p-tau231 antibody. Cleavage positions for trypsin are indicated with vertical lines and the identified peptides are indicated in black, while sequence portions not detected are in grey. Detected phosphorylated peptides are indicated. In (iii), the range of tryptic peptides identified in CSF immunoprecipitated using the p-tau231 antibody. In (iv), the range of tryptic peptides identified from GSK-3β-phosphorylated full-length tau-441, immunoprecipitated using the p-tau181 antibody. In (v) is shown the range of tryptic peptides identified in CSF immunoprecipitated using the p-tau181 antibody. In (vi) a schematic of full-length tau-441 with the different regions and indication of the epitopes of the antibodies used is shown. In (vii) is shown the range of tryptic peptides identified in CSF immunoprecipitated using the N-terminal antibody**.**

**Supplementary Figure 3. Plasma p-tau231 differentiates individuals with non-AD and AD dementia.** The AUC values of the ROC curves indicate the overall biomarker performance across groups, with 0.5 indicating no difference from chance and 1.0 a biomarker with specificity and sensitivity of 100%.

**
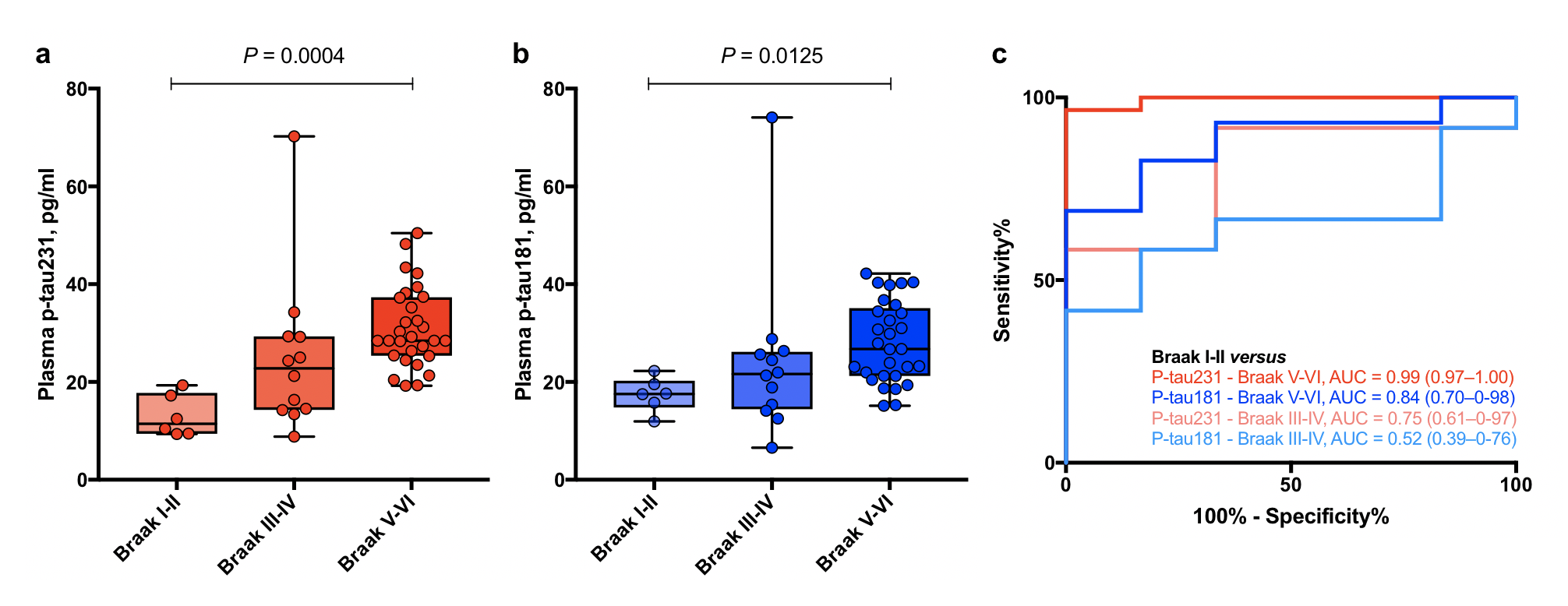
**

**Supplementary Figure 4. Plasma p-tau231 has a stepwise increase in Braak stages in neuropathologically confirmed cases.**

**
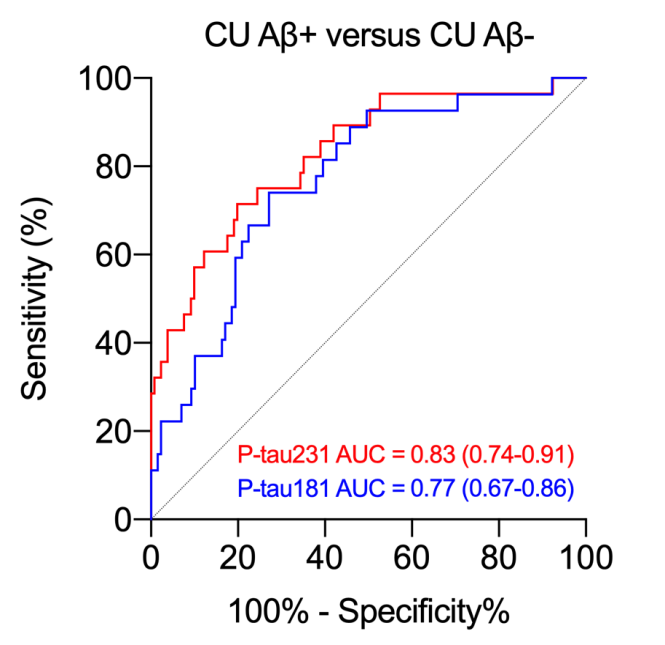
**

**Supplementary Figure 5. Plasma p-tau231 differentiates CU individuals who are Aβ-positive from CU individuals who are Aβ-negative.** The AUC values of the ROC curves indicate the overall biomarker performance across groups, with 0.5 indicating no difference from chance and 1.0 a biomarker with specificity and sensitivity of 100%.
